# Supplementary material for: HLA-A*24 Increases the Risk of HTLV-1-Associated Myelopathy despite Reducing HTLV-1 Proviral Load
Source: Int J Mol Sci. 2024 Jun 22;25(13):6858. doi: 10.3390/ijms25136858 (PMC11241684; doi:10.3390/ijms25136858)
Supplement: Supplementary file 1 [file ijms-25-06858-s001.zip › ijms-2956977-supplementary.pdf]

Supplemental Figure 1：Gating strategy in flow cytometry

A. Detection of HTLV-1 Tax-Pentamer positive cells

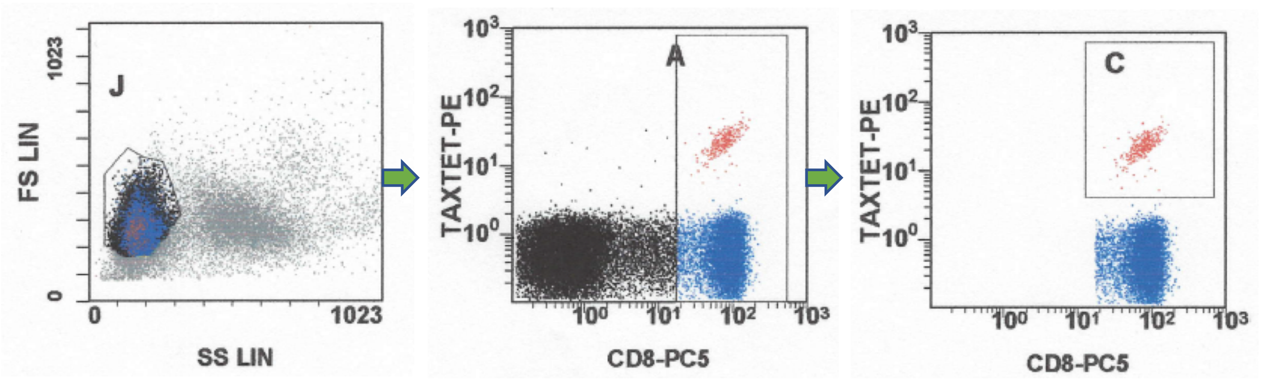

B. Detection of HTLV-1 Tax-specific IFN-γ positive cells

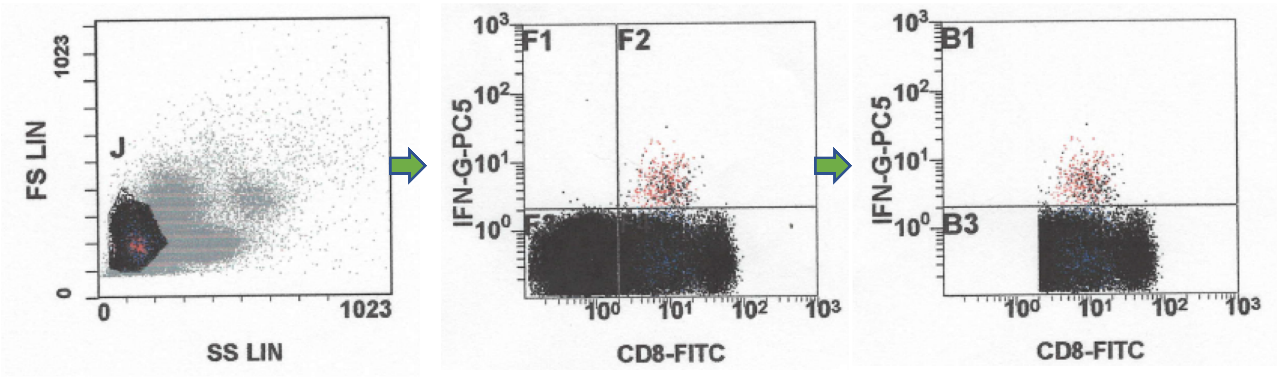

Supplemental Table 1. Clinical characteristics of HAM/TSP patients and ACs

	HAM (n = 152)	AC (n = 155)	*P* - value
Age	58.6	51.5	<0.001
Sex : Female/Male (Female %)	106 / 46 (69.7 %)	88 / 67 (56.8 %)	<0.001
HTLV-1 proviral load (PVL)	427 ± 452 *	252 ± 454 *	<0.001
Anti-HTLV-1 antibody (PA)	29057 ± 24982 *	4300 ± 7904 *	<0.001

* Mean ± S.D.
